# From analytical to data-driven: multi-method optimization of a bendable mirror system for dynamic focusing

**DOI:** 10.1107/S1600577526009422

**Published:** 2026-10-05

**Authors:** Baoning Sun, Zhixiang Wen, Qinghao Zhu, Jun Luo, Tairong Xu, Lingfeng Zhu, Yaxiang Liang, Jitao Sun, Zhuo Li, Shun Xia, Zhongmin Xu, Meiyi Wu, Qinming Li, Weiqing Zhang, Xueming Yang

**Affiliations:** a Dalian Coherent Light Source and State Key Laboratory of Chemical Reaction Dynamics Dalian Institute of Chemical Physics, Chinese Academy of Sciences Dalian116023 People’s Republic of China; b University of Chinese Academy of Sciences Beijing101408 People’s Republic of China; c Institute of Advanced Light Source Facilities, Shenzhen518107, People’s Republic of China; d Institute of High Energy Physics Chinese Academy of Sciences Beijing100049 People’s Republic of China; e Spallation Neutron Source Science Center, Dongguan523803, People’s Republic of China; Brazilian Synchrotron Light Laboratory, Brazil

**Keywords:** Kirkpatrick–Baez mirror, bendable mirror, transfer learning neural network, differential evolution algorithm, surface figure optimization

## Abstract

Accurate and efficient tuning of small-radius-of-curvature (RoC) bendable mirrors remains challenging under variable beamline conditions, while conventional analytical tuning is often inadequate. To address this problem, this study presents the design, modeling, and multi-method optimization of a small-RoC bendable mirror system developed as a preparatory study for the Shenzhen Superconducting Soft X-ray Free-Electron Laser. The system adopts a four-point bending structure with an asymmetric support layout, and integrated force sensors are used for direct real-time force monitoring. A physically grounded analytical route based on classical bending theory is first used to assess the basic deformation capability of the system. A data-driven route is then developed by combining a transfer learning neural network (TLNN) with a differential evolution (DE) algorithm. The TLNN surrogate model is trained on simulated force–profile pairs generated by finite element analysis and refined using matrix-style measured force-profile pairs. The results show that, within the investigated center RoC range of 450–700 m, the differences in fitted image distance and center RoC are maintained within 5 mm and 0.55 m, respectively. The root-mean-square height prediction errors are at the nanometre level. Furthermore, the TLNN–DE framework is able to recover the end-force configuration corresponding to a prescribed target surface profile. The deviation between the recovered and measured end forces is kept within 0.5 N. These results demonstrate that the proposed framework provides an effective and transferable route for data-driven optimization of bendable mirrors and other active or adaptive optical systems in free-electron laser and synchrotron radiation facility beamlines.

## Abstract 2

A small-radius-of-curvature bendable mirror system is developed for dynamic focusing in X-ray free-electron laser and synchrotron radiation beamlines, and a transfer-learning neural-network and differential-evolution framework is used for force-to-shape modeling and inverse optimization. The method achieves nanometre-level surface-profile prediction and sub-newton end-force recovery, providing an efficient route for rapid and repeatable tuning of active X-ray focusing mirrors.

## 1. Introduction

Advanced light sources, such as free-electron lasers (FELs) and synchrotron radiation facilities (SRFs), deliver photon beams with outstanding brightness, temporal and spatial resolution, and coherence (Buras & Materlik, 1986 ▸; Ackermann *et al.*, 2007 ▸; Emma *et al.*, 2010 ▸; Ishikawa *et al.*, 2012 ▸; Kang *et al.*, 2017 ▸; Wang, 2017 ▸; Zhao *et al.*, 2018 ▸; Decking *et al.*, 2020 ▸; Duris *et al.*, 2020 ▸; Prat *et al.*, 2020 ▸; Tanaka *et al.*, 2024 ▸), thereby enabling high-resolution imaging, spectroscopy, and diffraction experiments across a wide range of scales (Mimura *et al.*, 2010 ▸; Sakdinawat & Attwood, 2010 ▸; Cocco *et al.*, 2022 ▸). To preserve these source advantages at the sample position, the beamline must perform a series of essential optical operations, including beam transport, focusing, shaping, energy selection, and wavefront control (Xu *et al.*, 2023 ▸; Wu *et al.*, 2024 ▸; Sun *et al.*, 2025*a* ▸). Among the various beamline subsystems, the refocusing system is particularly important because it directly determines the beam quality and spatial resolution delivered to the sample position of the endstation. In this context, the Kirkpatrick–Baez (KB) mirror system, originally proposed in the 1940s, has become one of the most widely adopted solutions for X-ray focusing (Kirkpatrick & Baez, 1948 ▸). Its orthogonally arranged grazing-incidence mirrors provide high reflectivity, efficient beam convergence, and near-diffraction-limited focusing over a broad spectral range, which has established KB mirrors as a key technology in FEL and SRF beamlines as well as high-resolution X-ray microscopy. However, early KB systems were typically based on fixed-shape cylindrical mirrors, whose limited adaptability restricted their ability to satisfy diverse focusing requirements under changing experimental conditions.

This limitation has driven the development of dynamically bending mirror systems, in which the surface curvature can be actively adjusted to achieve tunable focal length and variable beam size (Dong *et al.*, 2007 ▸; Cocco *et al.*, 2010 ▸; Nistea *et al.*, 2017 ▸). Foundational theoretical studies by Padmore and Howells established the analytical basis for elliptical approximation by controlled end loading and laid the groundwork for subsequent engineering implementations (Howells *et al.*, 2000 ▸). On this basis, several representative technical routes have been developed. The Kirkpatrick–Baez Active Optics System (KAOS), implemented at FERMI and continuously refined over the past decade, demonstrated that a constant-width mirror substrate can realize effective active focusing through asymmetric end torques combined with wavefront sensing and feedback (Raimondi *et al.*, 2019 ▸; Manfredda *et al.*, 2022 ▸; Novinec *et al.*, 2024 ▸). In contrast, variable-width mirror substrates are designed with a trapezoidal geometry along the mirror length and have been developed for synchrotron applications, such as those at the European Synchrotron Radiation Facility (ESRF), to improve the approximation to the ideal elliptical figure through substrate-geometry tailoring (Zhang *et al.*, 2010 ▸). These developments have confirmed the value of dynamic bending for advanced beamline focusing, while also revealing an inherent trade-off between operational flexibility and figure-approximation accuracy. Variable-width designs are generally well suited to fixed beamline parameters, whereas constant-width rectangular substrates remain more attractive for flexible operation because of their structural simplicity and broader adaptability. At the same time, continued progress in mirror fabrication and bending mechanisms has significantly improved the achievable precision, stability, and reliability of dynamic focusing systems (Luo *et al.*, 2024 ▸), broadening their application in advanced light source facilities.

Despite these advances, small radius-of-curvature (RoC) dynamic bending remains a particularly challenging problem and deserves dedicated investigation. In FEL beamlines, dynamic focusing is required not only to maintain tightly focused beams but also to accommodate variations in source position, focal plane location, wavelength, and endstation configuration under different operating modes. This flexibility is often achieved by adopting rectangular mirror substrates, but this choice also leads to a larger deviation from the ideal elliptical figure as the required RoC decreases. At the same time, stronger bending drives the mirror closer to its material yield limit. In addition, the bending performance also depends on the reliability of the mechanical system. In practice, these factors make the mapping between the applied end loads and the resulting optical figure increasingly difficult to predict accurately. As a result, analytical estimation often provides a coarse starting point, whereas final optimization relies on iterative manual tuning and metrology feedback. This limitation becomes more pronounced when rapid and repeatable curvature retuning is required for flexible FEL operation. For this reason, there is a strong need for data-driven strategies to accurately model the bending response and address the inverse-control bottleneck of small-RoC bending systems.

To address the above challenges, this work presents the preliminary development and optimization of a bendable mirror system as a preparatory study for the Shenzhen Superconducting Soft X-ray Free-Electron Laser (S^3^FEL). S^3^FEL is a newly approved high-repetition-rate soft X-ray FEL facility under construction in China, designed to deliver 2.5 GeV electron beams at repetition rates up to 1 MHz and to generate radiation in the 1–30 nm wavelength range (Wang *et al.*, 2023 ▸). The bending mirror system adopts a four-point bending configuration with a modified support and loading structure. A multi-method optimization approach is implemented on this system, covering both analytical and data-driven strategies. The analytical method involves a coarse force estimation based on classical bending theory, followed by manual fine tuning. The data-driven method establishes a comprehensive modeling and optimization framework based on the transfer learning neural network (TLNN) and differential evolution (DE) algorithms. By combining simulation-based prior knowledge with limited measured-data refinement, this framework enables efficient surrogate modeling and inverse optimization for bendable mirror systems. Compared with pre-shaped or variable-width mirrors, bending a rectangular substrate from an initially flat state generally introduces a larger residual deviation from the ideal elliptical figure, particularly at small RoC. Nevertheless, the results show that the present bending system can generate smooth elliptical deformation with nanometre-level residual figure error. Ray-tracing simulations further indicate that this bending-induced residual is not the dominant limitation to the focusing performance under the investigated condition.

This paper is organized as follows. Section 2 introduces the basic theory, including elliptical bending theory, surface profile measurement based on a long trace profiler (LTP), and elliptical fitting. Section 3 describes the dynamic bending system, including the mechanical layout, the LTP instrumentation and environment, and the control architecture. Sections 4 and 5 present the analytical and data-driven optimization methods, respectively. Finally, Section 6 concludes the study.

## 2. Basic theory

### 2.1. Elliptical bending theory

**Figure 1:**
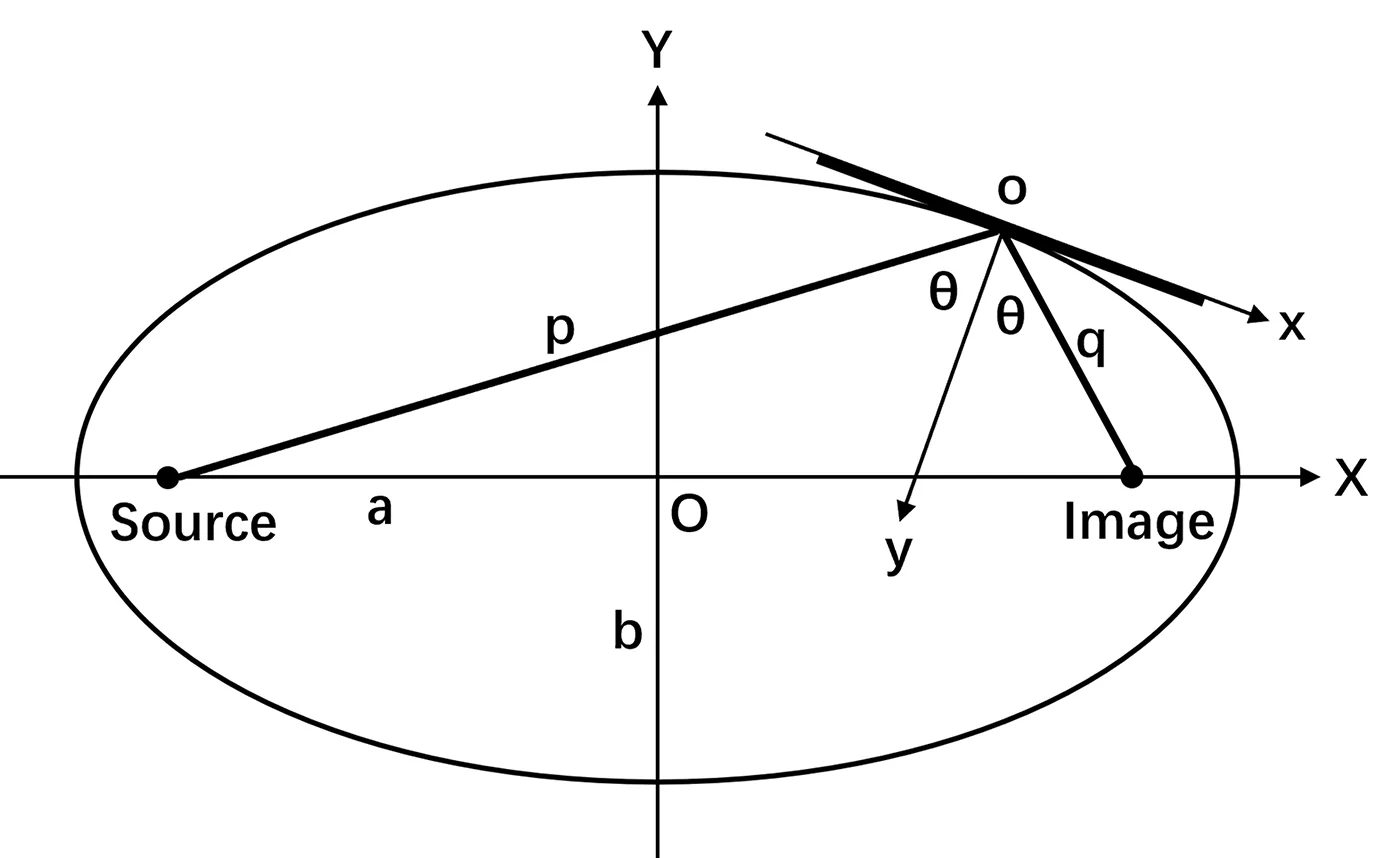
Schematic of the elliptical geometry for a KB mirror. The ellipse is defined by the object distance *p*, image distance *q*, and incidence angle θ. The local coordinate system is defined relative to the mirror surface.

To achieve high-quality X-ray focusing in a KB system, the mirror surface must approximate a meridionally elliptical profile defined by the beamline geometry. For bendable mirrors bent from initially flat substrates, this requirement can be realized by linking the ideal elliptical profile to the moment distribution along the mirror. The theoretical basis of this treatment is established from the geometrical properties of the ellipse and its local approximation using a low-order Taylor series expansion. Fig. 1 ▸ illustrates the elliptical geometry and the notation used to describe the mirror configuration.

The standard form of the ellipse is written as
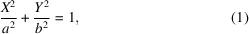
where *a* and *b* are the semi-major and semi-minor axes, respectively. In a local coordinate system defined on the mirror surface, the elliptical profile can be expressed by a power-series expansion,
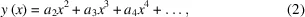
where the coefficients *a*_*i*_ correspond to specific aberration terms in the reflected wavefront. In particular, *a*_2_ describes defocus, *a*_3_ represents coma, and *a*_4_ contributes to spherical aberration. These coefficients are given by
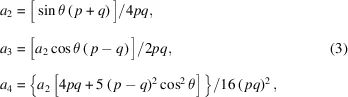
where *p* and *q* denote the source-to-mirror and mirror-to-image distances, respectively, and θ is the grazing incidence angle.

For bendable KB mirrors, a third-order approximation is generally sufficient to capture the dominant geometrical features required for preliminary micro-focusing design. The corresponding local slope and curvature are written as
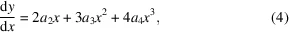

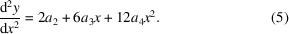
For an initially flat substrate bent into the required profile, the mirror figure is governed by the Bernoulli–Euler beam equation,
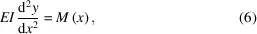
where *M*(*x*) is the bending moment distribution, *E* is Young’s modulus, and *I* is the second moment of area. When unequal end torques *C*_1_ and *C*_2_ are applied at the two ends of a mirror with length *L*, the bending moment varies linearly along the mirror axis,
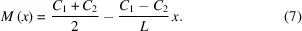
By matching this linear moment distribution to the second derivative of the third-order elliptical expansion, the end torques required for elliptical approximation can be obtained as
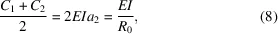

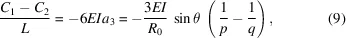
where *R*_0_ is the local RoC at the mirror center. These relations establish a direct connection between the optical design parameters and the mechanical loading conditions. Therefore, the third-order elliptical expansion provides a practical and analytically tractable basis for the design and optimization of bendable KB mirrors.

### 2.2. Elliptical fitting of LTP-reconstructed surface profiles

In the present work, the surface profile measurement is performed using an LTP and follows a standard numerical reconstruction procedure. Because the LTP directly measures the local surface slope rather than the surface height, additional processing is required before the measured data can be related to the optical design parameters of the mirror (Yashchuk, 2009 ▸; Siewert *et al.*, 2012 ▸). The slope data are first processed using the standard bidirectional scanning strategy to suppress first-order drift errors and improve measurement robustness. After removal of low-frequency alignment-related components, the slope data are numerically integrated to reconstruct the corresponding surface height profile.

To relate the reconstructed surface profile to the ideal optical geometry, elliptical fitting is performed to determine the effective image distance and to remove residual rigid-body offsets between the measured and theoretical profile coordinates. This procedure establishes a quantitative connection between the reconstructed mirror figure and the corresponding beamline parameters. In this fitting procedure, the object distance *p* and grazing-incidence angle θ are fixed at their nominal beamline values, whereas the image distance *q* is treated as the fitted optical parameter. The additional terms *h*_0_ and φ account for a constant height offset and a residual meridional profile tilt, respectively.

The theoretical elliptical profile is written as
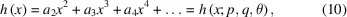
where *h*(*x*; *p*, *q*, θ) denotes the theoretical profile corresponding to the specified beamline geometry. By incorporating the height-offset and profile-tilt terms, the fitting expression becomes
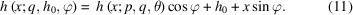
Given the reconstructed height data *h*_exp_(*x*_*j*_) at *N* sampling points *x*_*j*_, the optimal parameters *q*, *h*_0_, and φ are obtained by minimizing the least-squares objective function,
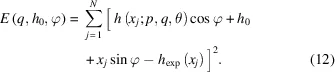
This nonlinear fitting problem is solved using the *lsqcurvefit* function in MATLAB, yielding the best-fit image distance, height offset, and tilt correction. The root-mean-square (RMS) value of the residuals is then calculated to quantify the fitting accuracy.

In addition to height-based fitting, direct fitting of the measured slope data *s*_exp_(*x*_*j*_) is also considered, which avoids the numerical integration step and provides an alternative estimate. In this case, the derivative of the elliptical profile is used as the fitting basis,
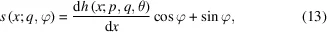
and the corresponding objective function is written as
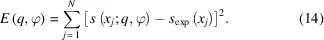
This slope-based fitting procedure returns the optimized values of *q* and φ, thereby providing a complementary evaluation of the effective optical geometry from the measured surface response.

## 3. System description

### 3.1. Mechanical layout

**Figure 2:**
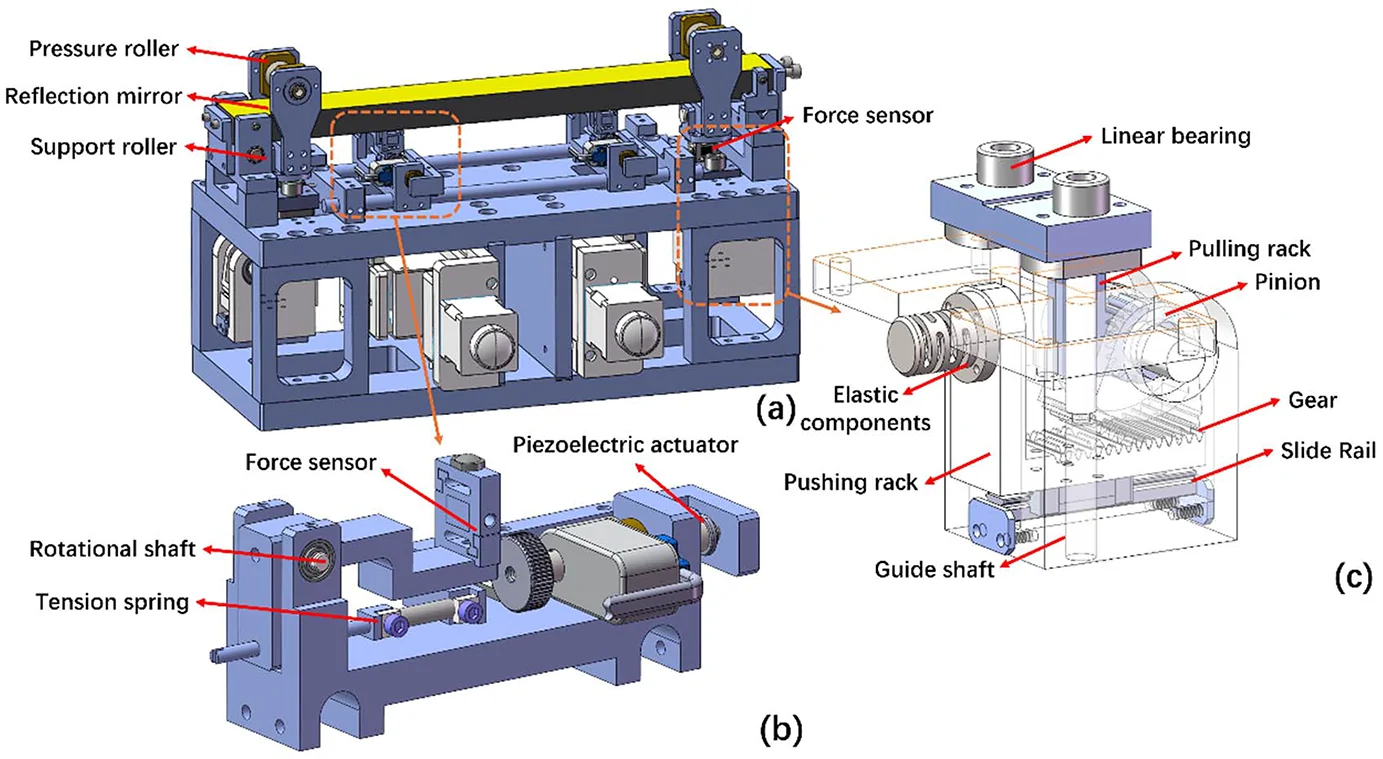
Mechanical configuration of the bendable mirror system. (*a*) Overall layout of the holder, showing the four-point bending structure and the integrated gravity-compensation units. (*b*) Schematic of the gravity-compensation unit. (*c*) Detailed view of a single pressure gear assembly.

A schematic of the mechanical holder for the bendable mirror is shown in Fig. 2 ▸. At the architecture level, the system consists of a primary four-point bending subsystem and an auxiliary gravity-compensation subsystem. The four-point bending geometry is defined by two passive support-roller contacts and two driven pressure-roller contacts. The two pressure-roller forces, denoted as *F*1 and *F*2, serve as the controllable primary bending inputs. The two gravity-compensation units are designed as auxiliary correctors for gravitational sag. The two pressure-roller contacts and the two gravity-compensation contacts are monitored by four force sensors, which provide contact-force feedback during operation. In the subsequent modeling and optimization analyses, only *F*1 and *F*2 are treated as independent inputs.

The mirror substrate is mounted horizontally with the reflecting surface facing upward and has dimensions of 400 mm × 40 mm × 20 mm (length × width × thickness). In the primary four-point bending subsystem, the mirror is supported at both ends while two pressure rollers apply downward forces near the ends to generate a controlled bending moment over the central region. To balance structural constraint with strain accommodation, an asymmetric support scheme is employed. One end of the mirror rests on a fixed support roller, whereas the other is supported by a rotational roller. This arrangement provides partial kinematic freedom for thermal and mechanical deformation and thereby reduces figure distortion during operation. Each pressure roller is coupled to the gear transmission mechanism through the corresponding force sensor and a pulling rack guided by linear bearings. The gear transmission mechanism adopts a rack–pinion–gear–rack configuration to convert motor thrust into the required bending actuation. In the initial design, a rack–gear–rack arrangement was considered for steering actuation. However, because the available motor thrust was insufficient for direct bending to the required curvature, a coaxial large-and-small gear mechanism is introduced to amplify the output force, as shown in Fig. 2 ▸(*c*). When the motor actuates the self-developed elastic component, the spring undergoes slight contraction and transfers the force vertically to the pushing rack. Constrained by the connected guide rail, the pushing rack is allowed to move only along a single axis and thereby drives a large gear simplified as a sector with one-sixth of a full gear to reduce mass. The motor provides a maximum thrust of approximately 100 N, whereas a force of at least 200 N is required to bend the mirror to the required curvature. To overcome this mismatch, a small pinion is mounted coaxially with the large gear, with a radius ratio of 1:2.5 between the pinion and the large gear. This configuration amplifies the motor output by a factor of 2.5, producing an effective force of 250 N, which is then transmitted to the pulling rack and ultimately applied to the mirror.

The auxiliary gravity-compensation subsystem is shown in Fig. 2 ▸(*b*). Each unit is fixed to the frame and consists of a forward-driven piezoelectric screw actuator that pushes a tensioning plate. The force is then transferred through a spring-loaded L-shaped lever rotating about a fixed axis and is applied upward to the underside of the mirror through a monitored contact point. In this manner, gravitational deformation can be compensated without disturbing the primary bending operation. The corresponding force signals are incorporated into the feedback loop to support closed-loop stabilization of the bending process.

### 3.2. Instrumentation and environment

**Figure 3:**
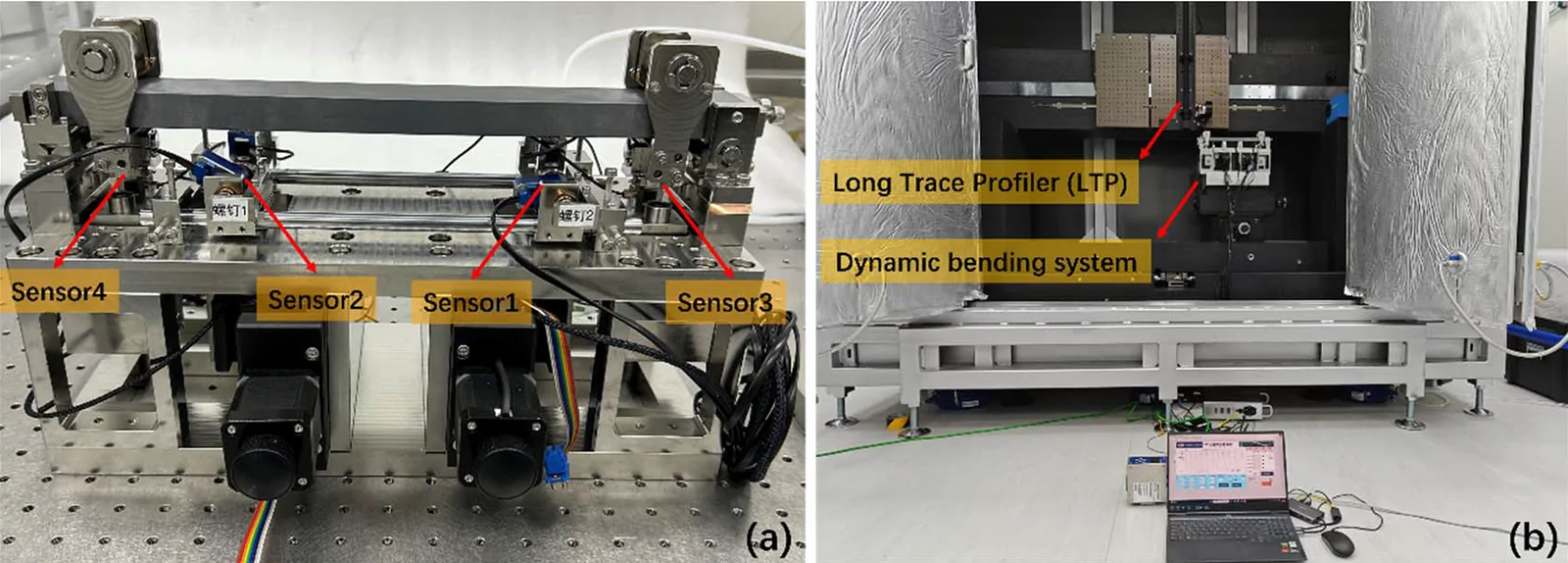
Prototype and measurement setup of the bendable mirror system. (*a*) Fabricated mechanical assembly with the mirror substrate installed in the holder and the locations of the four force sensors indicated. (*b*) Surface metrology of the bending system using an LTP in a cleanroom environment.

A prototype of the bendable mirror system is fabricated for experimental investigation. The assembled holder and measurement setup are shown in Fig. 3 ▸. Fig. 3 ▸(*a*) presents the fabricated stainless-steel mechanical structure, with an aluminium block temporarily placed at the intended mirror position during system integration and the locations of the four force sensors indicated. In this layout, the two primary bending forces *F*1 and *F*2 are measured by Sensor 3 and Sensor 4, respectively. All surface figure measurements are carried out in a cleanroom to minimize environmental disturbances. As shown in Fig. 3 ▸(*b*), the bending system is mounted on the granite base of an LTP for high-precision surface metrology. The LTP, developed in-house by the Spallation Neutron Source Science Center, adopts a flag-type scanning configuration. The LTP provides a slope reproducibility better than 15 nrad RMS, while its slope measurement accuracy for curved-mirror profiles is better than 50 nrad RMS. All measurements are performed in a thermally stabilized environment maintained at 20 ± 0.03°C over a continuous 24 h period to ensure measurement stability and repeatability.

### 3.3. Bending control

**Figure 4:**
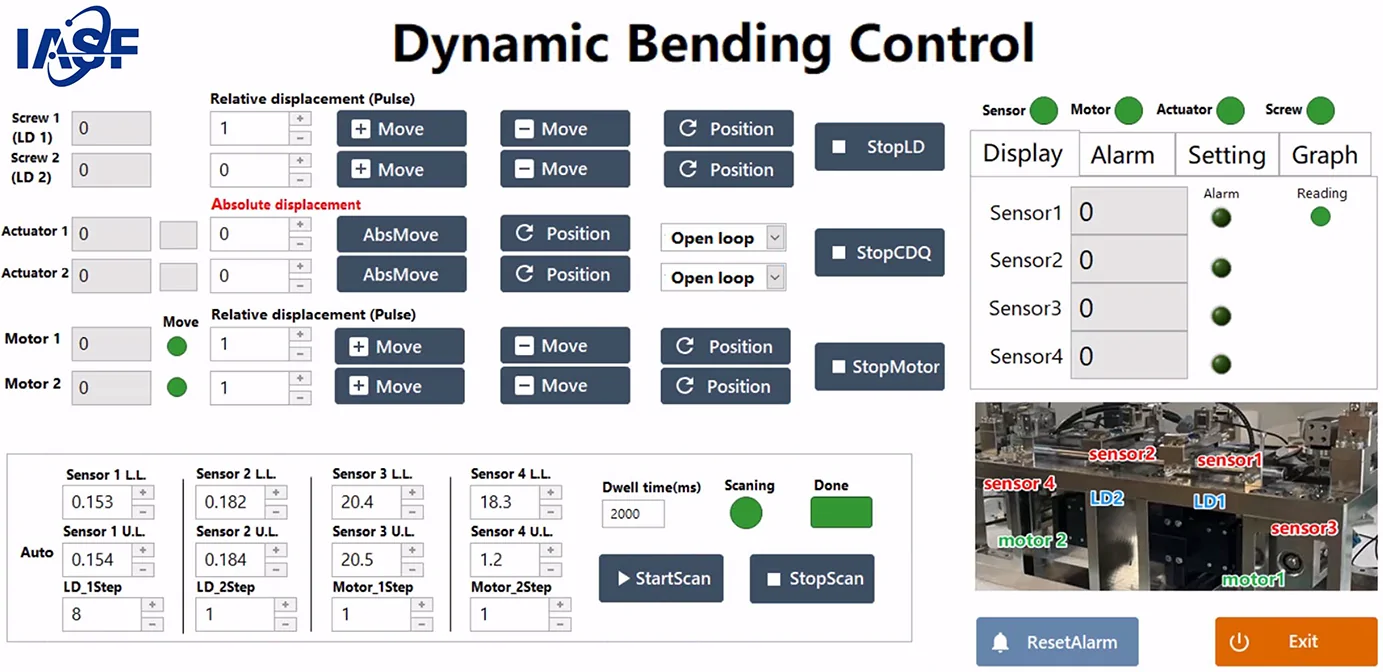
*LabVIEW*-based control interface of the bendable mirror system.

The bendable mirror system and the LTP-based surface metrology are operated in two independent *LabVIEW* control environments. The control interface for the bending system is shown in Fig. 4 ▸. It enables manual configuration and real-time monitoring of six hardware control channels, including ‘screw 1 (LD1)’ and ‘screw 2 (LD2)’ for gravity-compensation adjustment, ‘motor 1’ and ‘motor 2’ for the application of the primary bending forces through the pressure rollers, and ‘actuator 1’ and ‘actuator 2’ for fine force modulation. The screw motors are driven by relative displacement commands in pulse units, whereas the actuators are controlled by absolute displacement values.

The readings from the four force sensors are displayed in kilograms as equivalent gravitational forces and are used as real-time feedback for force tuning. In addition to manual operation, the control program supports automated matrix-based force scanning. Although the bending system and the LTP are controlled in separate environments, they can be coordinated through the automation routine to perform synchronized force loading and surface measurement. Upper and lower thresholds can be assigned independently to each scanned channel to define the scanning range and operational limits.

## 4. Analytical optimization with bending theory

### 4.1. Bending test under experimental configuration

To evaluate the bending performance under a representative micro-focusing condition, an experimental configuration consistent with the X-ray Photoelectron Spectroscopy (XPS) endstation of S^3^FEL is adopted. The beamline follows a two-stage focusing strategy, in which the upstream transport and monochromator optics define the effective source for the downstream KB refocusing system. The present bending mirror is considered as the vertical focusing element of this second-stage KB system. In this configuration, the bending mirror has an object distance of 47 m, an image distance of 5.1563 m, and a grazing-incidence angle of 17 mrad. The KB system is capable of achieving a focal spot with a full width at half-maximum (FWHM) of approximately 5 µm × 5 µm at an X-ray wavelength of 1 nm. In practical operation, the sample is positioned upstream of the focal spot so that a defocused beam is delivered at the sample position to obtain the beam size required by the experiment. Thermal-load and cooling effects are not considered in the present analysis.

**Figure 5:**
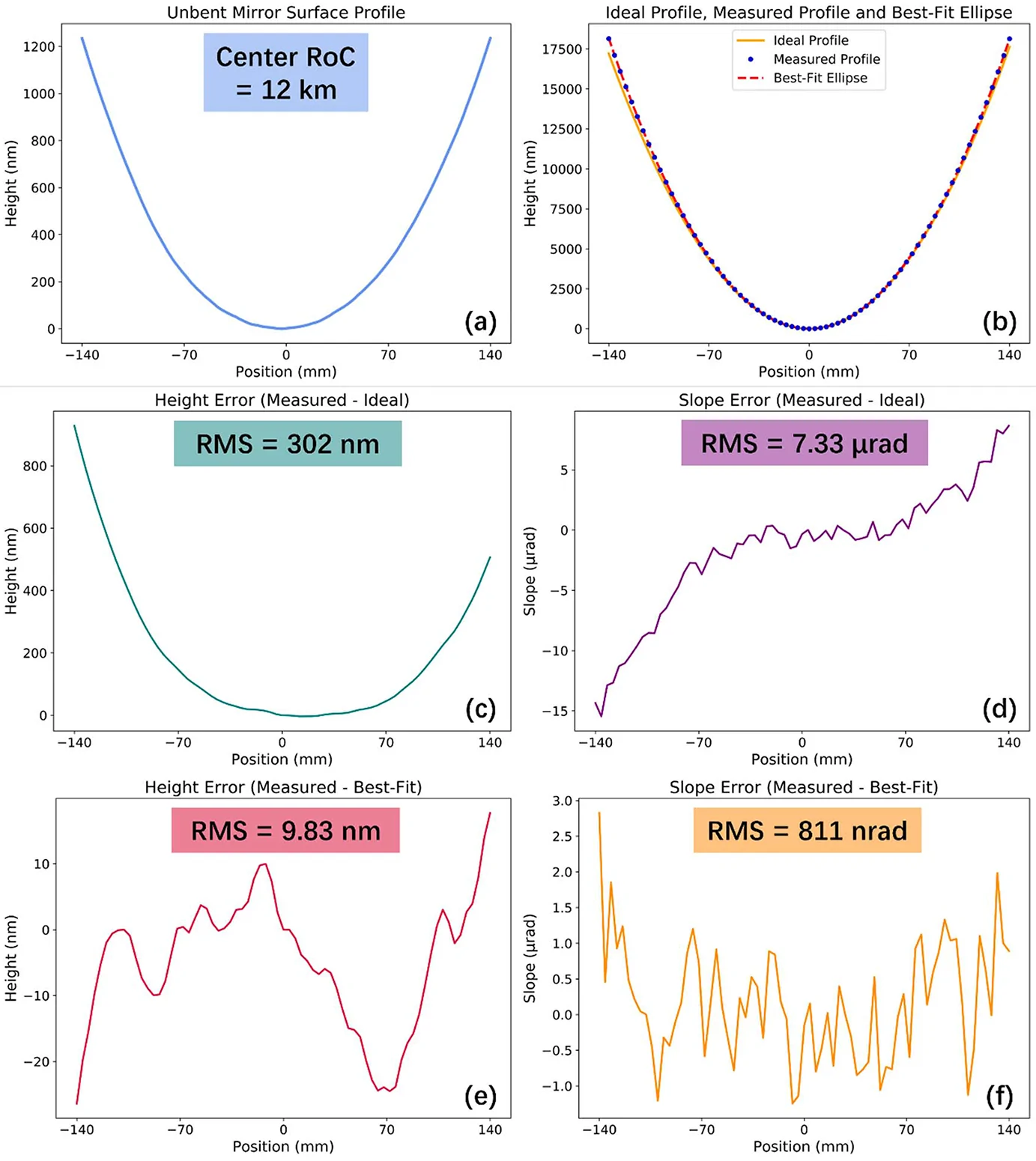
LTP measurements of the mirror surface. (*a*) Unbent mirror under gravity. (*b*) Measured surface profile under the experimental configuration, compared with the ideal profile and the best-fit ellipse. (*c*) Height error of the measured profile with respect to the ideal profile. (*d*) Slope error of the measured profile with respect to the ideal profile. (*e*) Height error of the measured profile with respect to the best-fit ellipse. (*f*) Slope error of the measured profile with respect to the best-fit ellipse.

To quantify the intrinsic deformation caused by self-weight, the mirror is first tested in the unloaded state, without applied bending force or activated gravity compensation. Surface profiles are measured by the LTP over the central 280 mm region with a spatial sampling interval of 1 mm, because partial obstruction from the bending holder limits the accessible measurement range to this central region. The resulting profile is shown in Fig. 5 ▸(*a*). A smooth and shallow sag is observed, which is attributed to gravitational deformation and can in principle be compensated through subsequent bending adjustment. The best-fit center RoC is approximately 12 km, indicating that the mirror remained effectively flat compared with the required RoC in the sub-kilometre range. On this basis, gravity compensation is not applied in the subsequent bending experiments, although the compensation unit remained mounted on the holder without contacting the mirror.

Surface measurements under the selected experimental configuration are then carried out over the same accessible region with a sampling interval of 4 mm. The end torques *C*_1_ and *C*_2_ are estimated from the analytical relations given in Section 2.1, with the mirror’s self-weight included, to approximate the ideal elliptical profile. The corresponding contact forces recorded by the two sensors are listed in Table 1 ▸. The measured results are summarized in Figs. 5 ▸(*b*)–5(*f*). Fig. 5 ▸(*b*) compares the measured surface profile with the ideal profile and the best-fit ellipse. Figs. 5 ▸(*c*) and 5 ▸(*d*) show the height and slope errors of the measured profile with respect to the ideal profile, whereas Figs. 5 ▸(*e*) and 5 ▸(*f*) show the height and slope errors after subtraction of the best-fit ellipse.

As shown in Fig. 5 ▸(*b*), the best-fit ellipse after bending has a center RoC of 552 m, corresponding to an image distance of 4.9457 m. These values differ from those of the ideal profile, which has a center RoC of 562 m and an image distance of 5.1563 m. The difference between the best-fit ellipse and the ideal profile yields an RMS height difference of 302 nm and an RMS slope difference of 7.33 µrad. For comparison, the theoretical residual caused by the uncompensated higher-order terms of the ideal two-end-force bending approximation is only 1.21 nm RMS in height and 39.3 nrad RMS in slope under the same optical condition. The much larger measured deviation observed in Figs. 5 ▸(*c*) and 5 ▸(*d*) is therefore attributed mainly to force-setting mismatch, assembly error, clamping- or support-induced distortion, and residual gravitational sag during the bending test.

After the best-fit ellipse is removed, the residual between the measured profile and the best-fit ellipse is 9.83 nm RMS in height and 811 nrad RMS in slope, as shown in Figs. 5 ▸(*e*) and 5 ▸(*f*). Its magnitude is comparable with the corresponding ellipse-fitting residual of the unbent mirror shown in Fig. 5 ▸(*a*), indicating that the bending process does not introduce clearly distinguishable additional figure degradation. This result should not, however, be interpreted as an improvement in the surface figure after bending. Instead, the residual remains dominated by the intrinsic surface error of the relatively coarse substrate, whose inherent slope error is around 1 µrad.

These results show that the analytical force estimate and manual tuning can generate a smooth elliptical deformation, but they are not sufficient to accurately match the target micro-focusing geometry in the present bending test. This limitation further motivates the introduction of a more efficient data-driven optimization route.

### 4.2. Ray-tracing simulations

**Figure 6:**
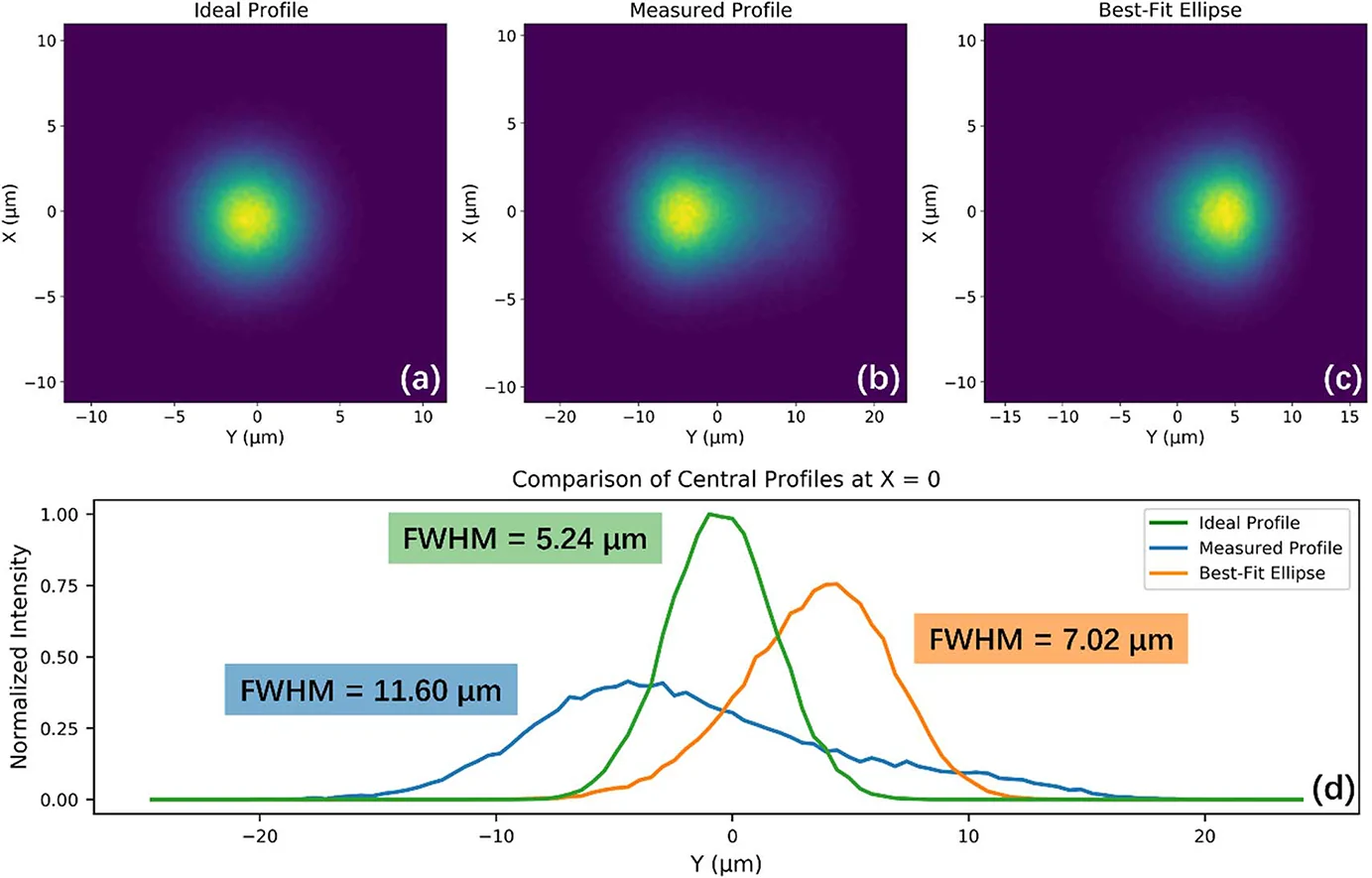
Ray-tracing simulation results for a 1 nm X-ray beam under the XPS endstation configuration. (*a*) Simulated focal spot obtained from the ideal profile. (*b*) Simulated focal spot obtained from the measured surface profile. (*c*) Simulated focal spot obtained from the best-fit ellipse. (*d*) Comparison of the vertical intensity distributions along the *Y* direction for the three cases.

Ray-tracing simulations were performed using the *SHADOW* ray-tracing module in the *OrAnge SYnchrotron Suite* (*OASYS*) software to evaluate the optical performance of the bendable mirror under the above experimental configuration (Rebuffi & Sanchez del Rio, 2016 ▸; Luca & Sanchez del Rio, 2017 ▸). In the simulated KB system, the bending mirror serves as the vertical focusing element, while the horizontal mirror is modeled as an ideal elliptical surface. Three surface profiles are assigned to the vertical mirror: the ideal profile, the measured profile, and the best-fit ellipse shown in Fig. 5 ▸(*b*). The corresponding simulation results are presented in Fig. 6 ▸. Figs. 6 ▸(*a*) to 6(*c*) show the simulated focal spots for the three cases, and Fig. 6 ▸(*d*) compares the intensity distributions along the vertical direction.

The ideal profile represents a standard elliptical surface that fully satisfies the prescribed design condition, and its focusing result can therefore be regarded as the upper performance limit of the present system. Under this reference condition, the measured profile produces a focal spot whose vertical FWHM is approximately twice that of the ideal case. This degradation highlights the influence of practical errors, including alignment inaccuracy, clamping-induced distortion, and force mismatch introduced during manual adjustment. By contrast, the best-fit ellipse exhibits substantially reduced mid- and high-frequency figure errors and yields a significantly narrower focal spot, much closer to the ideal focusing result. This comparison indicates that the current optical performance is not limited solely by the intrinsic capability of the bending scheme driven by two end forces to generate an elliptical figure but is also affected by the practical accuracy with which the ideal profile is realized. It also suggests that the focusing performance could be further improved by adopting optical substrates with higher initial figure quality, such as those manufactured by JTEC. Overall, the ray-tracing results show that, with the present bending scheme driven by two end forces and the manual tuning procedure, the system still has clear room for further improvement in focusing performance.

## 5. Data-driven optimization with neural network

### 5.1. Overview of the TLNN–DE framework

The analytical optimization approach based on bending theory provides a physically grounded starting point for mirror shape control. However, its practical implementation still relies heavily on iterative manual adjustment, and its efficiency becomes limited when frequent or rapid retuning is required. In realistic operation, deviations caused by alignment errors, fabrication tolerances, and mechanical limitations cannot be determined directly from the analytical solution alone. In contrast, data-driven methods based on machine learning provide an attractive alternative for describing complex system behavior without requiring a fully explicit analytical formulation (Gunjala *et al.*, 2023 ▸; Rebuffi *et al.*, 2023 ▸; Zhang *et al.*, 2025 ▸). Such methods are well suited to establishing efficient input–output mappings and have already shown strong potential in beamline optics and active control applications.

**Figure 7:**
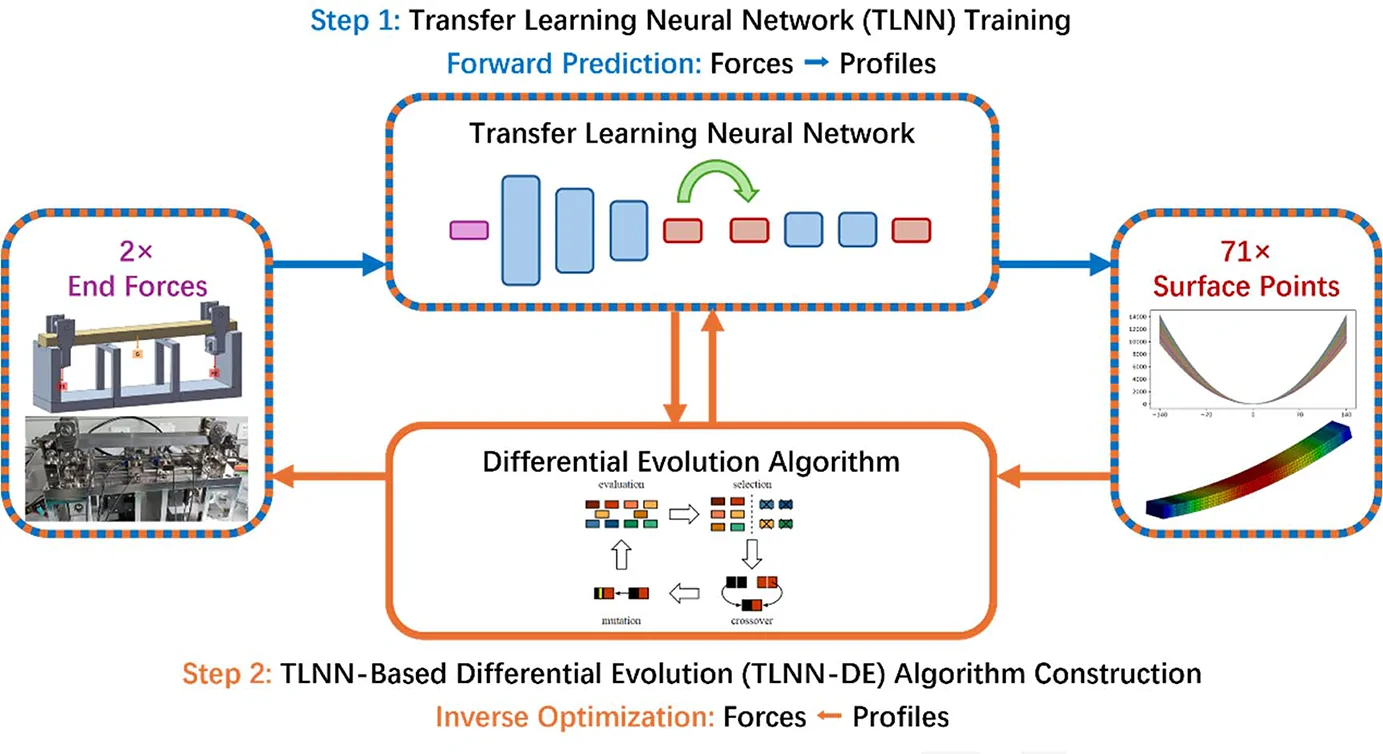
Workflow of the TLNN–DE optimization method for the dynamic bending system. A TLNN-based surrogate model is first constructed to perform forward prediction of surface profiles. A DE algorithm is then employed to recover the end forces corresponding to prescribed target profiles.

In this study, a two-stage data-driven framework is developed for surrogate modeling and inverse optimization of the dynamic bending system, as illustrated in Fig. 7 ▸. In the first stage, a TLNN is constructed as a surrogate model of the force-to-shape relationship. This stage includes two successive steps. A base model is first trained using a dataset generated from finite element simulations, containing 500 simulated force–profile pairs corresponding to randomly sampled end-force combinations. This simulated dataset provides broad coverage of the input space and allows the network to learn the fundamental deformation trend of the bending system. The pretrained model is then refined by transfer learning using 32 measured force–profile pairs, so that the surrogate can incorporate real-system characteristics and adapt to measurement uncertainties. By combining simulated and measured data, this strategy reduces the dependence on extensive LTP measurements.

In the second stage, the pretrained TLNN is coupled with a DE algorithm to form a hybrid TLNN–DE framework for inverse optimization. For a prescribed target surface profile, the DE algorithm iteratively queries the forward surrogate model to identify the end-force combination that best reproduces the target profile. This strategy transforms the inverse shape-control problem into an optimization process guided by a fast surrogate model, thereby improving both efficiency and accuracy under realistic operating conditions. More broadly, the proposed framework provides a practical route for data-driven control of dynamic bending systems and may also be extended to other active or adaptive optical systems for which accurate analytical descriptions are difficult to establish.

### 5.2. Simulation dataset generation by finite element analysis

**Figure 8:**
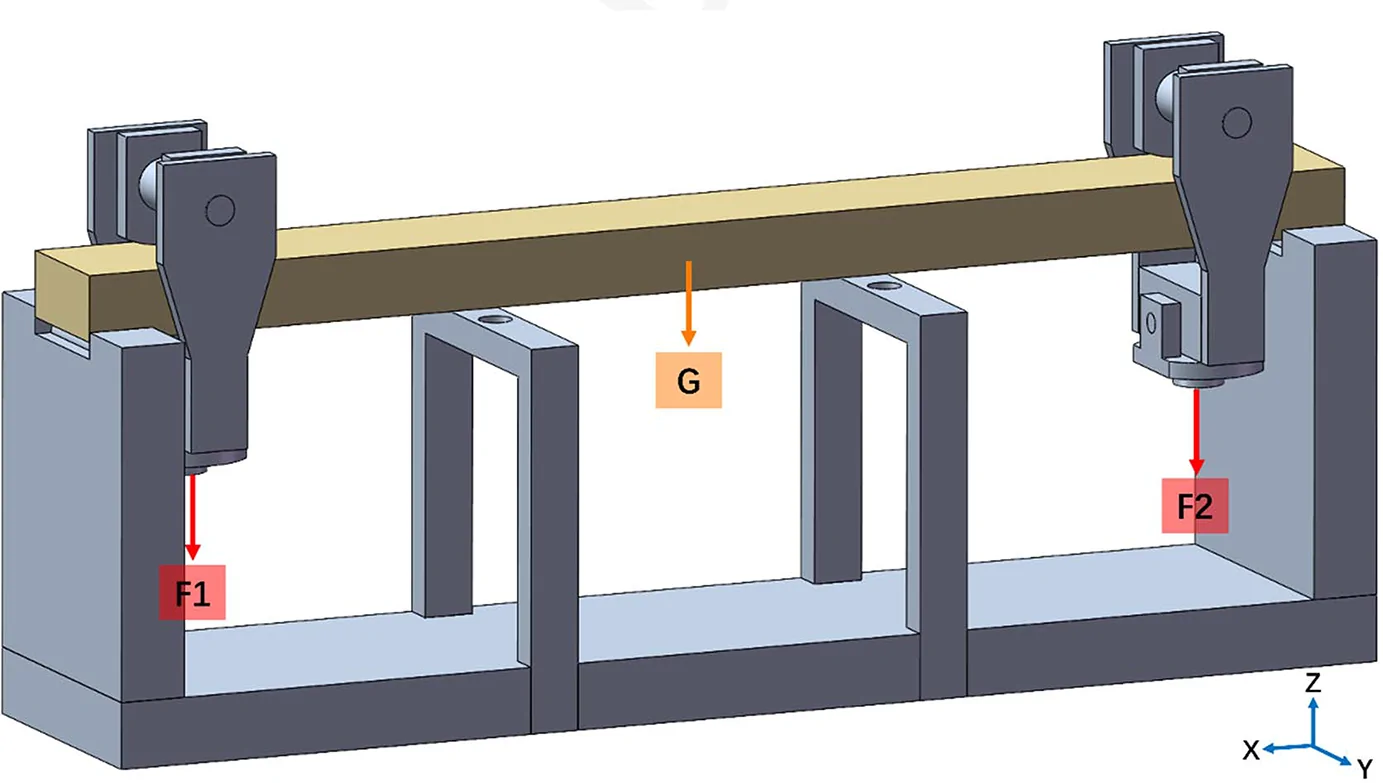
Simplified finite element model of the bending mechanism. End forces are applied at both ends of the mirror, and standard earth gravity is included in all model components.

To provide an initial dataset for surrogate-model training, a simplified finite element model of the bending mechanism is constructed in *ANSYS Workbench*, as shown in Fig. 8 ▸ (Ansys, 2023 ▸). In this model, two end forces (*F*1 and *F*2) are applied to generate the bending moments at the two ends of the mirror, while the gravity-compensation mechanism is omitted. Standard earth gravity (*G*) is applied uniformly to all model components. The material parameters used in the simulation are listed in Table 2 ▸.

The finite element model is intentionally simplified because the purpose of this simulation stage is not to reproduce every mechanical detail of the holder but to capture the dominant deformation behavior of the bending system over the full force range of interest. For this reason, a relatively coarse mesh is adopted to improve data-generation efficiency while retaining the main force-to-shape response. The element body size is set to 5 mm, and the contact region size is set to 2 mm. Under this configuration, the computation time is reduced from approximately 20 min to less than 3 min per case, which makes large-scale dataset generation practical. A total of 500 simulated surface profiles are generated using randomly sampled end-force combinations in the range 150 N to 250 N. The dataset size is selected to provide sufficient coverage of the input space for base-model training at acceptable computational cost. The entire simulation workflow is automated through scripting in *ANSYS Workbench*, and all cases are completed within one day on a workstation equipped with an Intel Xeon Gold 6240 CPU (2.6 GHz).

**Figure 9:**
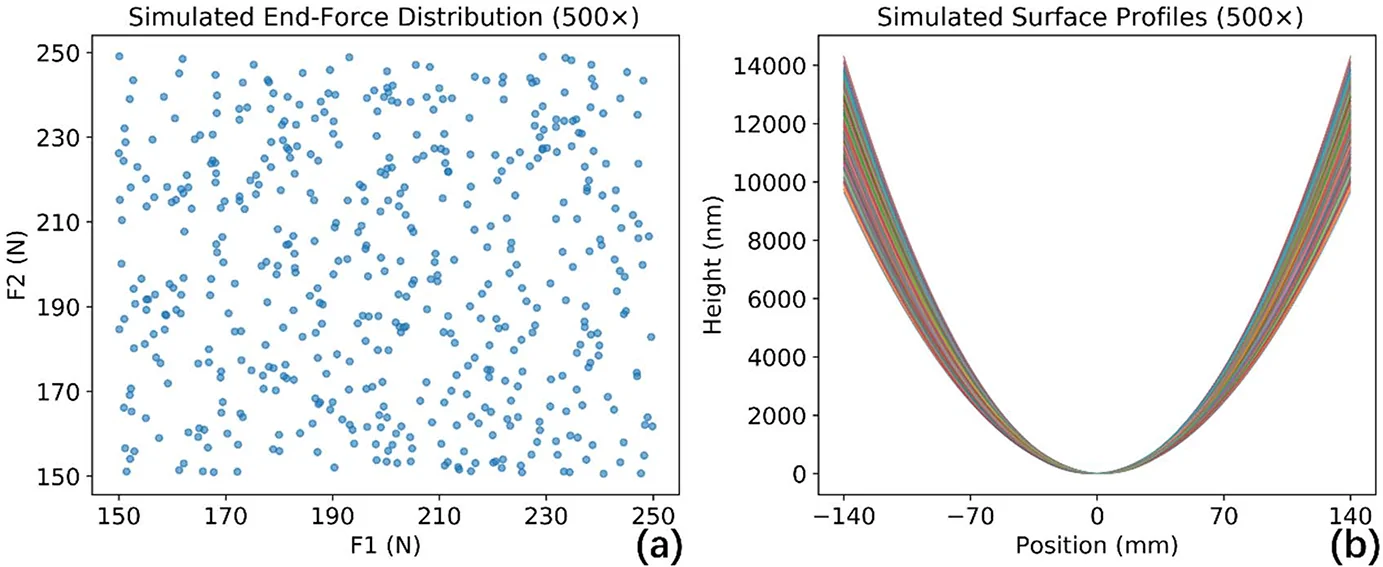
Simulated dataset for base model training. (*a*) Distribution of 500 randomly sampled end-force combinations. (*b*) Corresponding simulated surface profiles.

To ensure consistency with subsequent measured profiles, the simulated surface profiles are uniformly resampled to the same accessible central 280 mm measurement region with a spatial interval of 4 mm. The simulated dataset is shown in Fig. 9 ▸. Fig. 9 ▸(*a*) presents the distribution of the sampled end forces, and Fig. 9 ▸(*b*) shows the corresponding simulated surface profiles. This dataset serves as the basis for learning the global deformation trend of the dynamic bending system before refinement with measured data.

### 5.3. Matrix-style measured dataset acquisition for model refinement

The simulated dataset provides only an idealized description of the mirror deformation behavior under simplified boundary conditions. To incorporate the real-system mechanical response of the bending system, a matrix-style measured dataset is acquired for model refinement. The measurement matrix is designed to span the same end-force range used in the finite element simulations, namely 150–250 N, so that the measured data can directly complement the simulated dataset within a consistent input domain. During the experiment, the force values are read in kilograms from the integrated load sensors and then converted into newtons using a gravitational acceleration of 10 m s^−2^ to maintain consistency with the simulation data.

A total of 36 end-force combinations are initially arranged in a 6 × 6 matrix with 20 N intervals between adjacent force levels. Owing to signal-transmission errors during the coordinated operation of the bending-control program and the LTP measurement system, four measured profiles are excluded, yielding 32 valid force–profile pairs for model refinement. In addition, ten independent force cases are measured separately as test samples. Among them, five cases are used to evaluate the forward prediction capability of the surrogate model, whereas the other five are reserved for subsequent inverse-optimization assessment. Each measurement requires approximately 20 min, and the total acquisition time is about 14 h.

**Figure 10:**
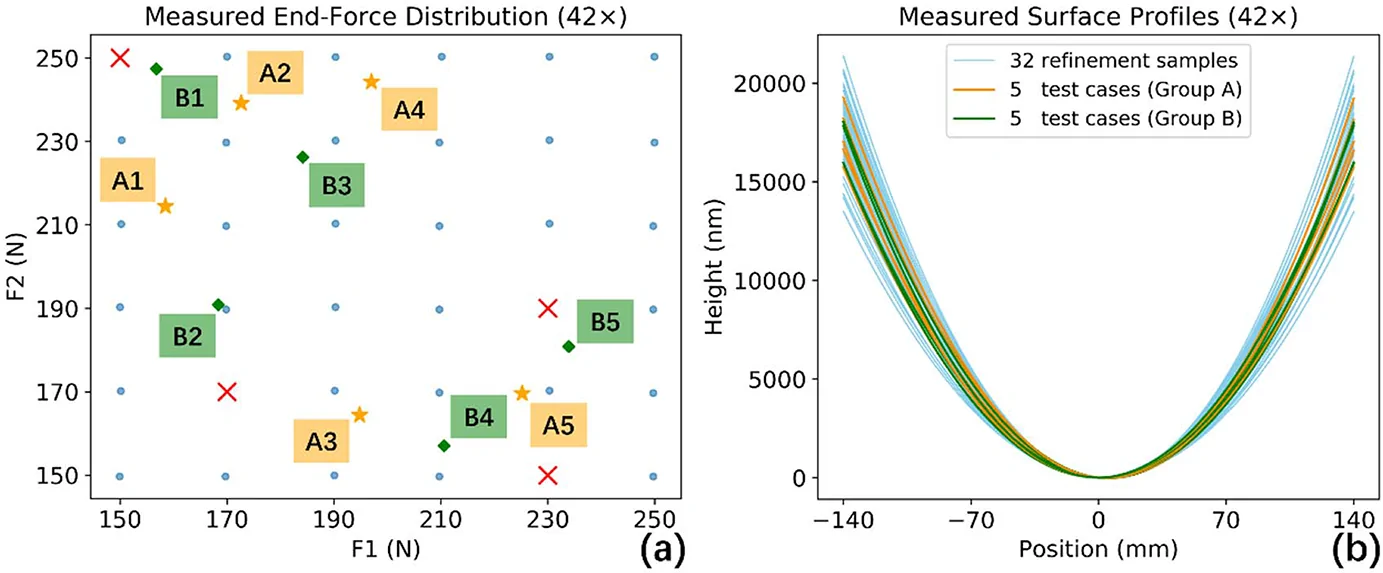
Matrix-style measured dataset used for model refinement and independent testing. (*a*) Distribution of applied end-force combinations. Blue circles denote the 32 refinement samples, orange pentagrams denote the five forward-prediction test cases, and green diamonds denote the five inverse-optimization test cases. (*b*) Corresponding measured surface profiles for all cases.

The distribution of the measured dataset is shown in Fig. 10 ▸. Fig. 10 ▸(*a*) presents the applied end-force combinations, where the refinement samples are marked in blue, the forward-prediction test cases in orange, and the inverse-optimization test cases in green. The corresponding measured surface profiles are shown in Fig. 10 ▸(*b*). The two groups of test samples are labeled A1–A5 and B1–B5, respectively, and their acquisition order is randomized to examine repeatability and robustness under practical operating conditions. The specific end-force combinations of these independent test cases are listed in Table 3 ▸. The measured profiles exhibit a peak-to-valley (PV) range of 14–22 µm, which is higher than the 10–14 µm range obtained from the simulation dataset. This difference reflects the presence of systematic offsets and real-system deviations that cannot be predicted directly from the analytical or simplified simulation models alone. As a result, the refinement stage does not merely improve numerical fitting accuracy, but also compensates for the gap between analytically guided simulation and the real-system mechanical behavior of the bending system.

### 5.4. Transfer learning-based surrogate model

**Figure 11:**
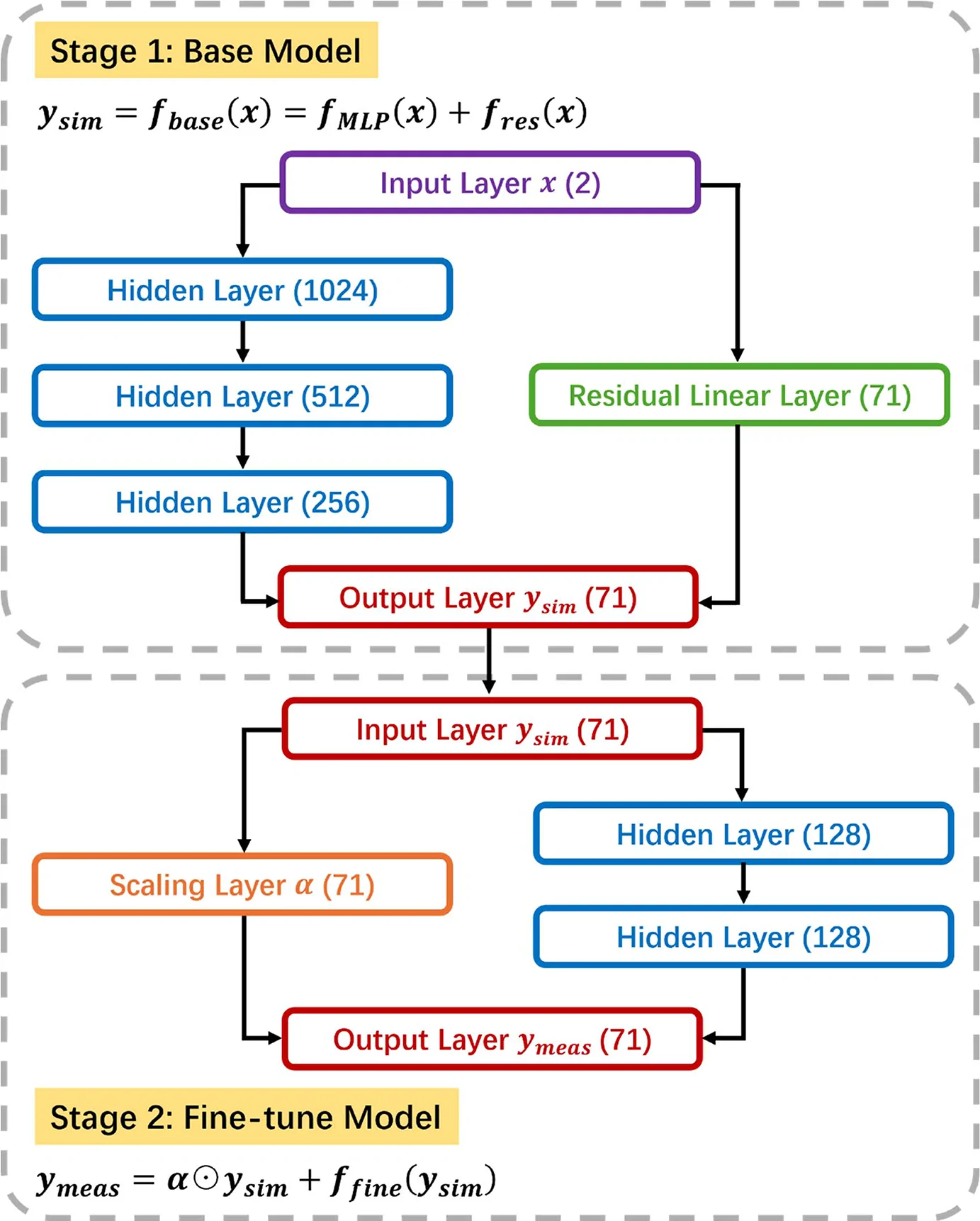
Two-stage architecture of the TLNN surrogate model. In Stage 1, the base model predicts simulated surface profiles from end-force inputs through a multilayer network with a residual linear branch. In Stage 2, a trainable scaling vector and a residual correction network are introduced to refine the base-model prediction using measured data.

To establish a surrogate model that combines simulated and measured data, a TLNN is implemented in Python using the *PyTorch* framework (Ansel *et al.*, 2024 ▸). The TLNN is designed as a two-stage surrogate-modeling framework, as illustrated in Fig. 11 ▸.

In Stage 1, a base model *f*_base_ is constructed to learn the fundamental mapping from end forces to simulated surface profiles. The network takes the two-dimensional end-force vector *x* as input and outputs a 71-dimensional simulated surface profile *y*_sim_. The main branch, denoted as *f*_MLP_(*x*), consists of three fully connected hidden layers with dimensions of 1024, 512, and 256. Each hidden layer includes a linear transformation, batch normalization, a sigmoid-weighted linear unit (SiLU) activation, and a dropout layer with a dropout rate of 0.4. In parallel with the main branch, a residual linear layer, denoted as *f*_res_(*x*), maps the two-dimensional input directly to the 71-dimensional output. The final output of Stage 1 is obtained by summing the nonlinear prediction and the residual linear term, which preserves the direct dependency between input forces and surface profiles and improves model generalization. The output of the base model is expressed as
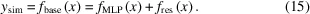
In Stage 2, the pretrained base model is refined through a lightweight fine-tuning model *f*_fine_ to incorporate measured-data correction. A trainable 71-dimensional scaling vector α is first applied element-wise to *y*_sim_ to adjust the overall amplitude of the base-model prediction. In parallel, a residual correction network takes *y*_sim_ as input and predicts the remaining discrepancy between the simulated and measured profiles. This correction network contains two hidden layers, each with 128 neurons and SiLU activation functions, followed by a 71-dimensional output layer. The final TLNN-predicted profile *y*_meas_ is written as
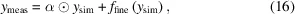
where ⊙ denotes element-wise multiplication. In this way, the fine-tuning stage corrects the base-model prediction through both global scaling and local residual compensation.

For training, all force inputs and profile outputs are normalized to the range [−1, 1] using shared scaling coefficients. Stage 1 is trained on the simulated dataset with a batch size of 64 for 5000 epochs using the Adam optimizer and the mean squared error (MSE) loss. After Stage 1, the base-model parameters are frozen. Stage 2 is then trained on the measured refinement dataset for 5000 epochs, during which only the scaling vector α and the residual correction network are updated. In this stage, the AdamW optimizer, SmoothL1 loss, cosine-annealing learning-rate schedule, and gradient clipping are adopted to improve training stability. The training time is approximately 5 min for Stage 1 and 1 min for Stage 2 on a laptop equipped with an Intel Core i7-10875H CPU (2.30 GHz) and an NVIDIA GeForce RTX 2070 GPU, with the model training mainly accelerated by the GPU. For model retention, the Stage 1 model state with the lowest training loss on the simulated dataset is preserved, whereas in Stage 2 the model state achieving the lowest root-mean-squared error (RMSE) on the measured refinement dataset is retained for subsequent evaluation.

### 5.5. Forward prediction performance of the TLNN

**Figure 12:**
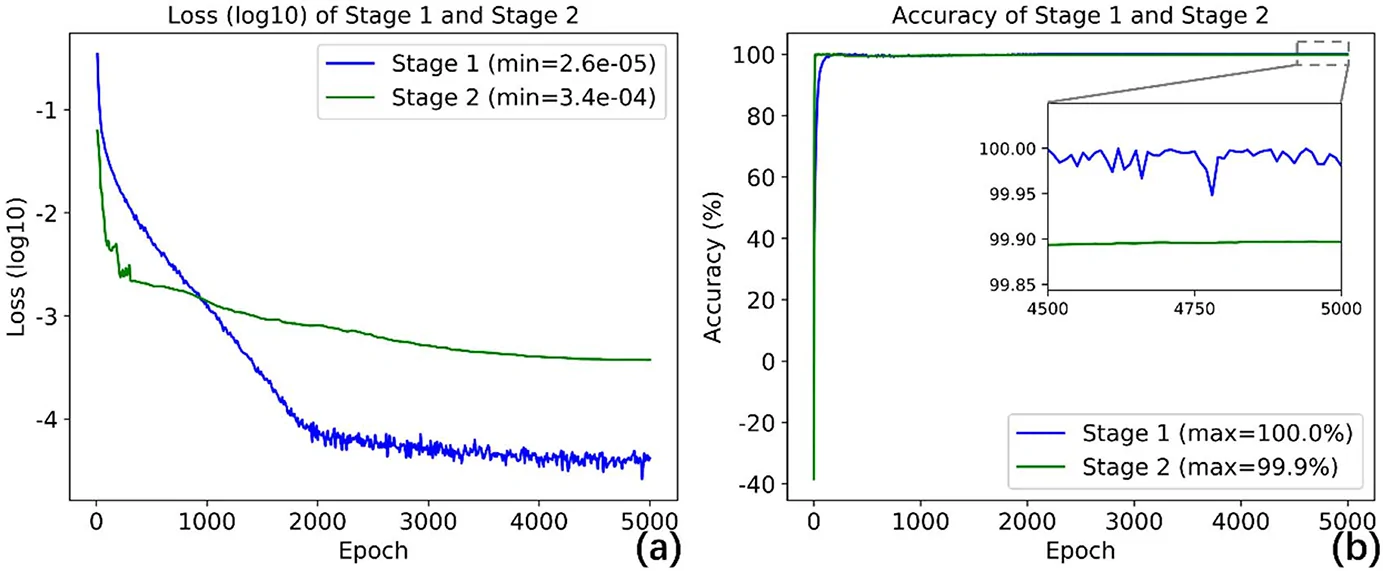
Training behavior of the TLNN in the two-stage learning process. (*a*) Training loss curves for Stage 1 and Stage 2. (*b*) Corresponding prediction accuracy curves during the training process.

The forward prediction performance of the TLNN is first evaluated from a theoretical perspective based on the training loss and prediction accuracy, as shown in Fig. 12 ▸. In Stage 1, the network is trained using the mean squared error (MSE) loss, which quantifies the deviation between predicted and reference surface profiles,
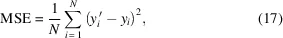
where *y*_*i*_ denotes the surface height at the *i*th sampling point from the dataset, 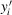 is the corresponding network prediction, and *N* is the total number of sampling points in one surface profile. In Stage 2, a more complicated SmoothL1 loss is adopted during refinement to improve robustness to measurement noise and local deviations in the measured dataset. Because different loss functions are used in the two stages, the curves in Fig. 12 ▸(*a*) are presented mainly to illustrate the convergence trend of each training stage rather than for direct quantitative comparison. As shown in Fig. 12 ▸(*a*), the loss curves in both stages decrease progressively and exhibit stable convergence during training. In both cases, the models have already entered a sufficiently converged regime by approximately 5000 epochs.

As a supplementary evaluation metric, prediction accuracy is defined as
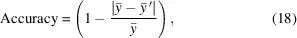
where 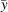 and 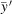 represent the mean surface-height values of the reference and predicted profiles, respectively. As shown in Fig. 12 ▸(*b*), the prediction accuracy remains close to 100% throughout both training stages, reaching 100.0% in Stage 1 and 99.9% in Stage 2. Together with the converged loss behavior, this result indicates that the TLNN provides a stable and consistent surrogate model of the dynamic bending system.

**Figure 13:**
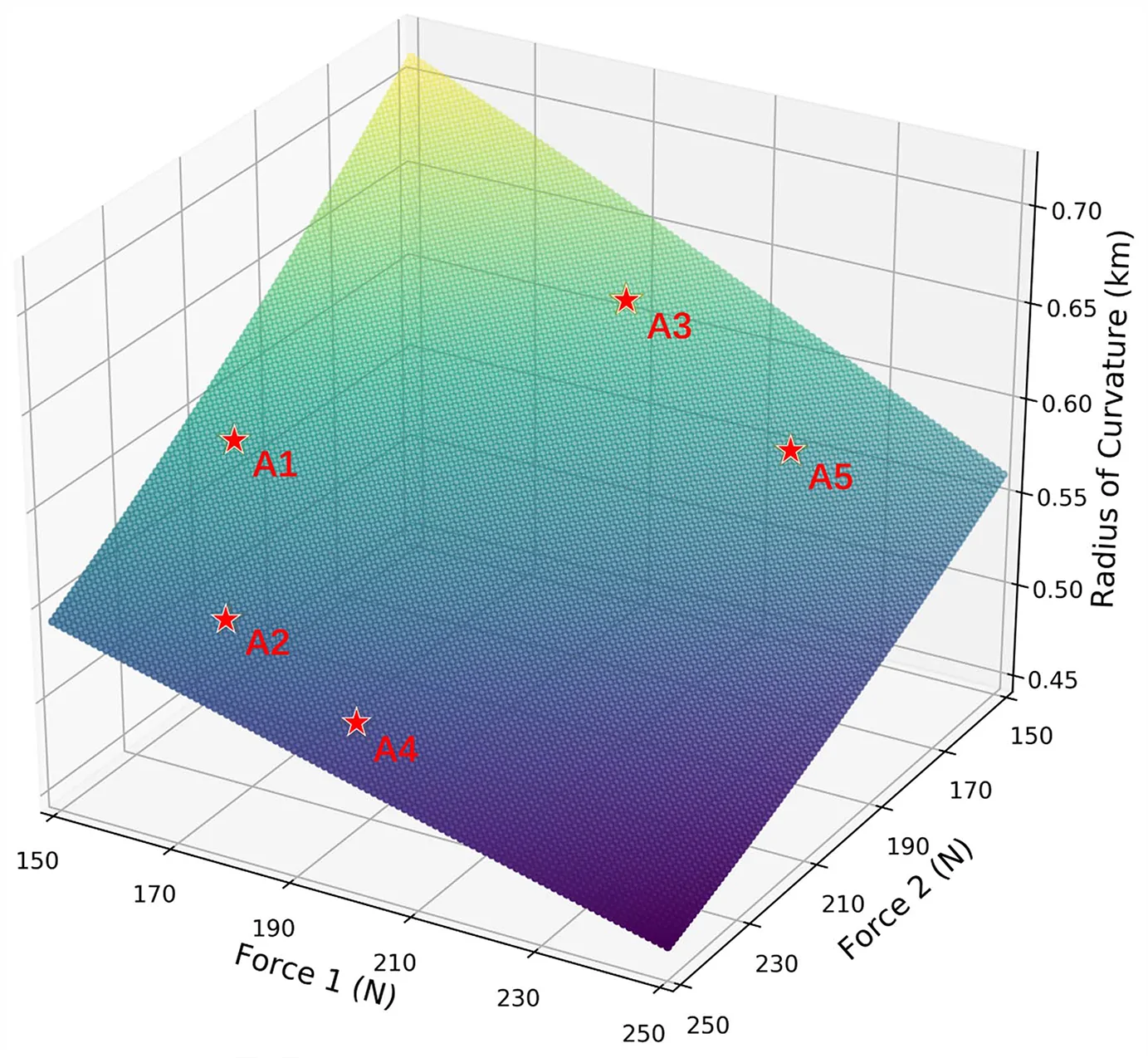
Center RoC map fitted from TLNN-predicted surface profiles over the *F*1–*F*2 end-force input domain. The orange pentagrams mark the five independent forward-prediction test cases A1–A5.

To further examine the global prediction behavior of the trained surrogate model, a two-dimensional map of the fitted center RoC is constructed over the *F*1–*F*2 input domain, as shown in Fig. 13 ▸. The fitted RoC values span approximately 450–700 m, indicating that the model captures the wide-range curvature modulation capability of the dynamic bending system under asymmetric end-force control. This range reflects the present dataset coverage rather than the full design capability of the system, for which the expected center RoC range is 0.3–12 km. The five independent forward-prediction test cases A1–A5 are overlaid as orange pentagrams in Fig. 13 ▸, and their detailed parameter comparisons are summarized in Table 4 ▸. The differences between the center RoC values fitted from the measured and TLNN-predicted profiles are all below 0.55 m.

**Figure 14:**
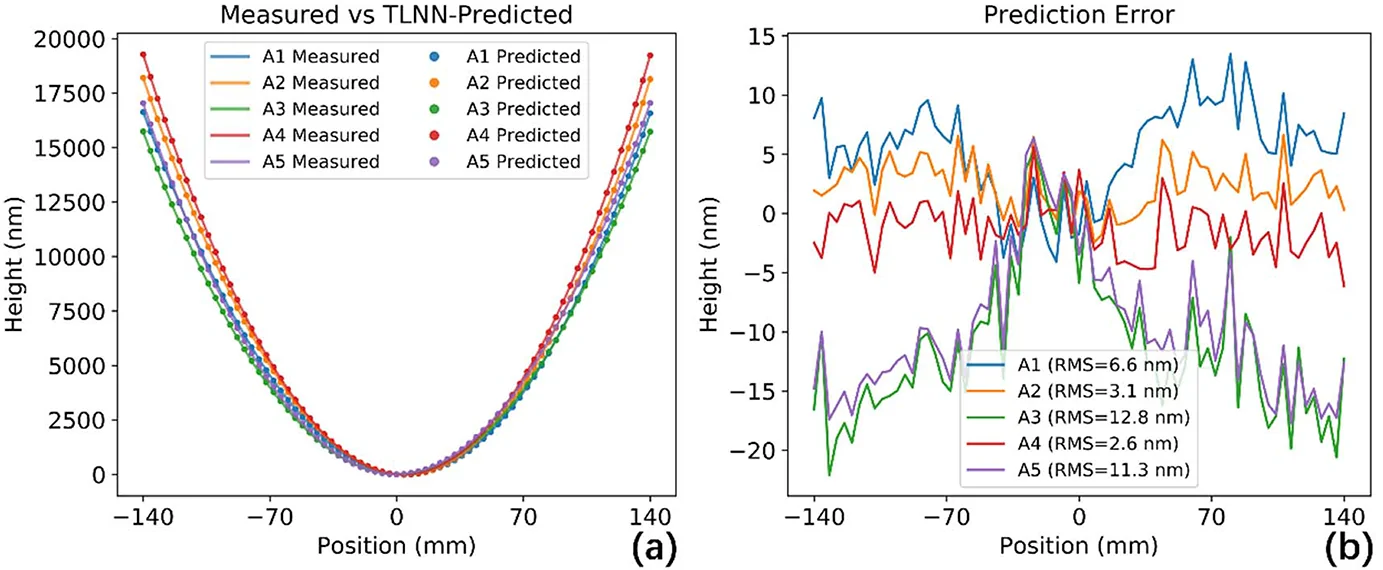
Forward-prediction performance of the TLNN evaluated on the five independent test cases A1–A5. (*a*) Comparison between TLNN-predicted and measured surface profiles. (*b*) Corresponding prediction errors.

To gain further insight into the profile-level predictive performance, the five independent forward-prediction test cases A1–A5 are examined in detail. The TLNN-predicted surface profiles are compared with the corresponding measured profiles in Fig. 14 ▸(*a*), and the associated prediction errors are shown in Fig. 14 ▸(*b*). In all five cases, the TLNN-predicted profiles agree closely with the measured profiles. The residuals exhibit two opposite quadratic-like patterns, with samples A1, A2, and A4 showing one pattern and samples A3 and A5 showing the other. This behavior suggests slight systematic differences between local measurement regions, most likely associated with small variations in the mechanical clamping condition. These systematic residuals are not eliminated by further increasing the training epochs, and their amplitudes remain on the order of 10 nm in all cases. For comparison, the pointwise RMS repeatability of the curved-profile measurement under repeated loading at the same force is approximately 2–3 nm. Moreover, the remaining residuals can also be partly attributed to the finite prediction accuracy of the trained TLNN. The Stage-2 model achieves a profile-prediction accuracy of about 99.9%, corresponding to a relative error of about 0.1%, which is equivalent to approximately 7–9 nm RMS height error for the tested curved profiles. Therefore, these residuals can be regarded as negligible for practical forward prediction. Overall, these results confirm that the TLNN accurately captures the force-to-shape relationship of the bendable mirror under realistic operating conditions.

The predictive capability of the TLNN is also evaluated at the level of focusing parameters. In addition to the center RoC discussed above, Table 4 ▸ further compares the image distances fitted from the measured and TLNN-predicted profiles. For all five forward-prediction test cases, the differences in fitted image distance remain within 5 mm. These results demonstrate that the TLNN provides not only accurate profile-level prediction but also a reliable pre-adjustment estimate of the effective focusing geometry. Nevertheless, additional fine-tuning at the experimental station is still expected in practical operation, with guidance from external diagnostics such as screen-based spot monitoring, wavefront sensing, or similar feedback tools.

### 5.6. Differential evolution for inverse optimization

Although the forward TLNN accurately captures the deformation behavior of the bending mirror, the ultimate goal of the present framework is to solve the inverse problem, namely to determine the end-force configuration required to reproduce a prescribed target surface profile. A direct inverse model based on a simple neural network was tested, but the results were unsatisfactory, with an accuracy of only about 80% and excessively large RMS errors. This limitation arises from the intrinsic non-uniqueness of the inverse mapping, because similar surface profiles may correspond to different end-force combinations. Under such conditions, a purely data-driven inverse network does not easily converge to a stable and reliable solution. To overcome this limitation, the pretrained forward TLNN is coupled with a DE algorithm to form a hybrid inverse-optimization framework. In this scheme, the DE algorithm iteratively searches the input space, while the TLNN serves as a fast evaluator of the corresponding surface profile. This hybrid strategy follows the general DE-based global optimization framework (Price *et al.*, 2005 ▸) and is consistent with recent active-optics optimization approaches that combine DE with neural-network surrogate models (Sun *et al.*, 2025*a* ▸; Sun *et al.*, 2025*b* ▸). This combination enables efficient identification of end-force configurations with both rapid convergence and high fidelity to the target profile.

**Figure 15:**
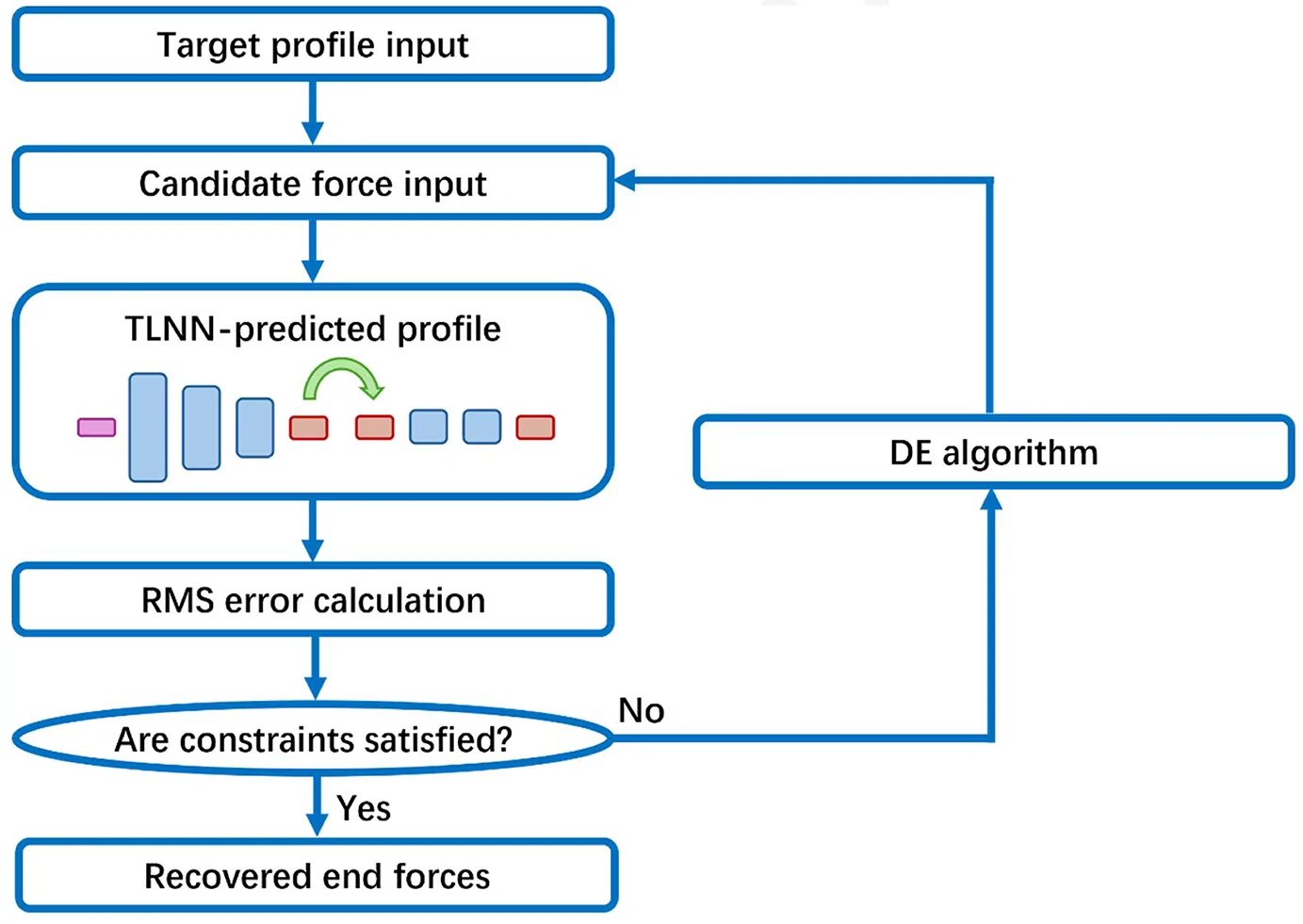
Workflow of the TLNN–DE framework for inverse optimization to recover the end-force combination corresponding to a prescribed target surface profile.

The workflow of the TLNN-DE framework is shown in Fig. 15 ▸. Starting from a pretrained TLNN surrogate model and a prescribed target surface profile, the DE algorithm initializes a population of candidate end-force vectors within the allowable range. Each candidate is evaluated by the TLNN, which predicts the corresponding surface profile. The optimization objective is defined as the pointwise RMS deviation between the TLNN-predicted surface profile and the target surface profile (Vannoni *et al.*, 2014 ▸; Vannoni *et al.*, 2015 ▸). The DE algorithm then updates the candidate end-force values iteratively to minimize this objective function. The iteration is terminated when either the maximum number of generations is reached or the objective value falls below a predefined threshold. The final solution gives the recovered end-force combination that best reproduces the target profile.

This optimization framework is not limited to end-force control of the present bending system. It can also be extended to other actuation schemes, such as voltage-driven piezoelectric deformable mirrors and power-controlled resistive heating mirrors (Zhang *et al.*, 2015 ▸; Alcock *et al.*, 2019 ▸; Cocco *et al.*, 2020 ▸; Xu *et al.*, 2024 ▸; Sun *et al.*, 2025*a* ▸; Sun *et al.*, 2025*b* ▸; Nistea *et al.*, 2025 ▸). In addition, the DE-based formulation allows explicit constraints to be imposed on the optimization variables, including upper and lower bounds as well as inter-variable relationships. Region-specific target profiles can also be defined when localized shape control is required. Importantly, the DE optimization is fully decoupled from the TLNN training stage, which is based on finite element simulations and measured-data refinement. This separation enables fast execution during deployment and makes the framework practical for real-time or near-real-time control applications.

### 5.7. Inverse optimization performance of the TLNN–DE framework

The TLNN–DE algorithm is implemented in Python using the Geatpy library (Jazzbin, 2020 ▸), and the key optimization parameters are summarized in Table 5 ▸. The allowable end-force range is set to [150 N, 250 N], consistent with the input domain used in the TLNN surrogate model. The measured surface profiles of the five independent inverse-optimization test cases B1–B5 are sequentially assigned as target profiles, and the corresponding end-force configurations are determined by the optimization algorithm. For all five cases, convergence is achieved within 0.2 s on the same laptop specified in Section 5.4, with the DE-based inverse search mainly performed on the CPU, which indicates the high computational efficiency of the proposed framework.

**Figure 16:**
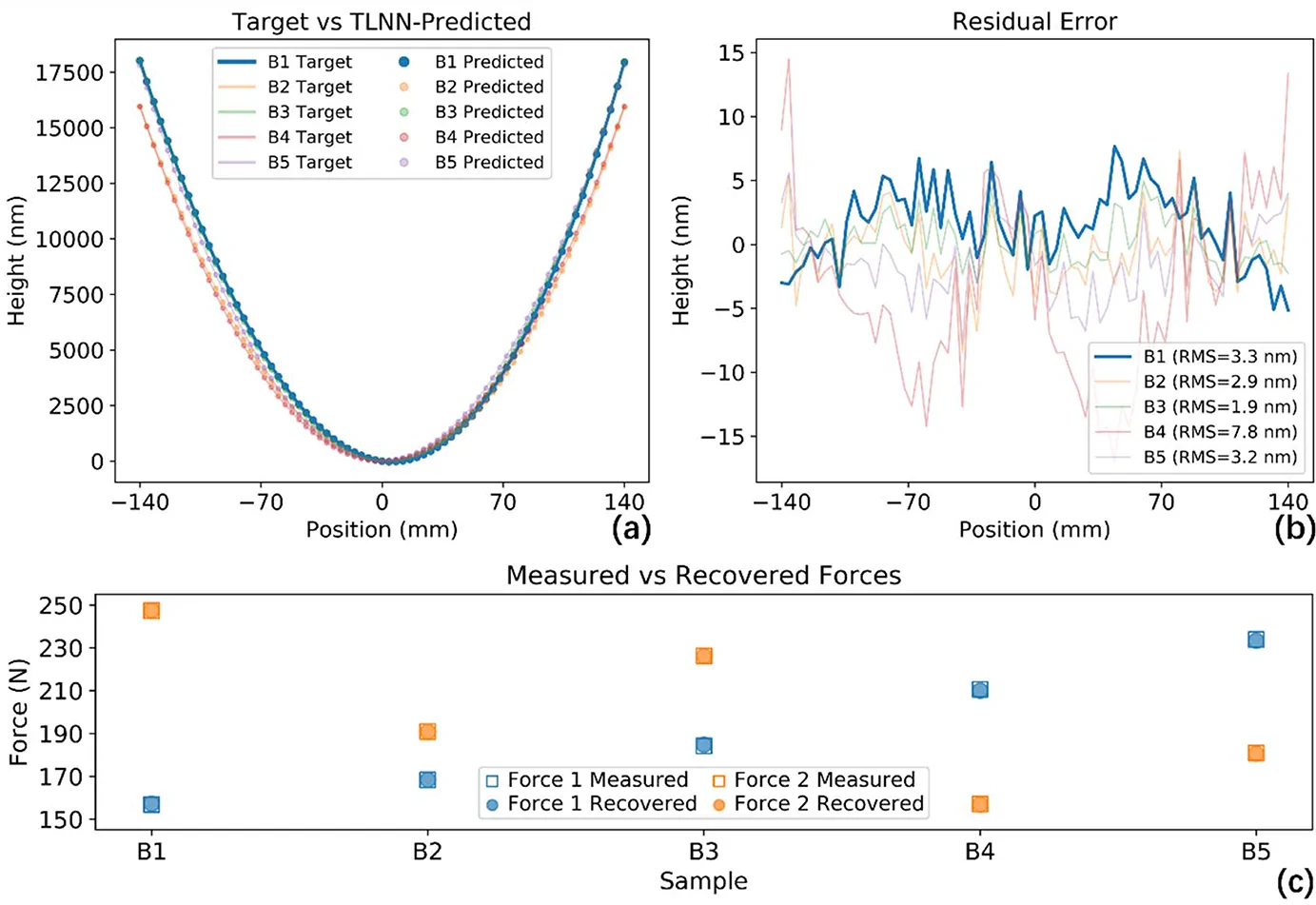
Inverse-optimization results obtained by the TLNN–DE framework for the five independent inverse-optimization test cases B1–B5. (*a*) Comparison between the TLNN-predicted surface profiles obtained from the recovered end-force inputs and the target profiles. (*b*) Corresponding residual errors. (*c*) Comparison between the recovered and measured end-force combinations.

The overall inverse-optimization results are presented in Fig. 16 ▸. Fig. 16 ▸(*a*) compares the TLNN-predicted surface profiles obtained from the recovered end-force inputs with the corresponding target profiles, and Fig. 16 ▸(*b*) shows the associated residual errors. In all five cases, the TLNN-predicted profiles agree closely with the target profiles, and the residual errors remain at the nanometre level. These results demonstrate that the TLNN–DE framework can accurately identify end-force combinations that reproduce the prescribed target profiles. Sample B1 is highlighted in both subfigures as a representative case. Its residual profile exhibits both medium- and high-frequency components, which may arise from numerical artifacts and from uncompensated higher-order terms in the bending system. The recovered end-force configurations are summarized in Fig. 16 ▸(*c*). For all five inverse-optimization test cases, the deviation between the recovered and measured end forces remains within 0.5 N. This result indicates that the TLNN–DE framework recovers the corresponding actuation inputs with excellent consistency. Such behavior confirms the robustness of the surrogate model during inverse optimization, even in the presence of measurement uncertainty and mechanical imperfection. More importantly, it shows that the proposed framework can efficiently identify practical input solutions for a non-unique inverse problem that is difficult to solve accurately through purely analytical estimation and manual tuning.

The use of different validation metrics in the forward-prediction and inverse-optimization assessments reflects the different objectives of the two tasks. The forward-prediction assessment focuses on whether the TLNN can accurately map end forces to surface profiles and preserve the corresponding effective focusing geometry, whereas the inverse-optimization assessment focuses on whether a prescribed target profile can be reproduced by the recovered end-force inputs. To further provide a comparable optical-level evaluation, the image distance and center RoC are also fitted from the target profiles and from the TLNN-predicted profiles obtained using the recovered end-force inputs. For the five inverse-optimization test cases, the maximum difference in fitted image distance is 5.3 mm, and the differences in fitted center RoC remain below 0.49 m. This consistency is reasonable because the DE algorithm does not rely on an independently trained inverse neural network, but searches the force space using the trained forward TLNN as a fast profile evaluator. Overall, these results demonstrate that the proposed TLNN–DE framework provides an effective inverse-optimization route for dynamic bending systems.

## 6. Conclusion and perspective

This study presents the preliminary development and multi-method optimization of a small-RoC bendable mirror system through both analytical and data-driven approaches. The analytical method, based on elliptical bending theory and manual force adjustment, provides a physically grounded starting point for mirror shape control and confirms the basic deformation capability of the holder. However, the surface figure and ray-tracing results show that analytical estimation combined with manual tuning is insufficient to accurately drive the system to the intended optical condition. The main limitation arises from practical factors such as alignment error, clamping-induced distortion, and force mismatch during adjustment. This limitation motivates the introduction of a more efficient data-driven optimization framework.

To address this problem, a TLNN–DE framework is established for surrogate modeling and inverse optimization of the dynamic bending system. The TLNN is trained in two stages. In Stage 1, a base model is constructed using 500 simulated force–profile pairs generated by automated finite element analysis in *ANSYS Workbench*. In Stage 2, the pretrained model is refined using 32 matrix-style measured force–profile pairs. Five additional independent cases are used for forward-prediction evaluation, and another five are reserved for inverse-optimization assessment. The results show that the TLNN provides stable convergence during training and accurately captures the force-to-shape relationship of the bending system. For the forward-prediction test cases, the prediction errors remain on the order of 10 nm, while the differences in fitted image distance and center RoC remain within 5 mm and 0.55 m, respectively. These results confirm that the surrogate model can reliably predict the surface profile and determine the effective focusing geometry.

On this basis, the pretrained TLNN is coupled with a DE algorithm to solve the inverse problem of determining the end-force configuration corresponding to a prescribed target profile. The inverse-optimization results show that convergence is achieved within 0.2 s for all test cases, the TLNN-predicted profiles obtained from the recovered forces agree with the target profiles at the nanometre level, and the deviations between the recovered and measured end forces remain within 0.5 N. These results demonstrate that the TLNN–DE framework can provide accurate and efficient inverse solutions for the dynamic bending system under realistic experimental conditions. More importantly, they confirm that the proposed data-driven route effectively overcomes the practical limitations of purely analytical estimation and manual adjustment for this class of non-unique inverse-control problems.

The present work establishes a transferable modeling and optimization framework for small-RoC bendable mirrors and, more broadly, for active or adaptive optical elements in FEL and SRF beamlines. In practice, this suggests that future bendable mirrors could be calibrated before installation and then deployed with minimal on-site tuning. At present, however, the LTP-based metrology and optimization correspond to a fixed upstream optical geometry defined by *p*, *q*, and θ, for which surface-figure metrology is sufficient. In realistic beamline operation, these optical parameters are more likely to vary with the working condition. This limitation motivates extension of the present framework from surface-figure prediction to beam-oriented optimization by incorporating optical performance metrics such as focal spot size and wavefront quality through optical simulation or experimental measurement. In parallel, a water-cooling module will be introduced to investigate thermal deformation and performance stability under coupled cooling, clamping, and loading conditions. Long-term stability and corresponding correction strategies will also be studied systematically. Ultimately, this framework aims to support the implementation of closed-loop adaptive control for real-time surface figure correction in high-performance optical systems.

## Figures and Tables

**Table 1:** Contact forces recorded during the bending test

Type	Sensor ID	Measured force (N)
End bending	Sensor 3 (*F*1)	218.4
	Sensor 4 (*F*2)	188.0

**Table 2:** Material parameters used in the finite element simulation

Material	Density (kg m^−3^)	Young’s modulus (GPa)	Poisson’s ratio
Si	2330	169	0.3
Stainless steel	8000	206	0.33

**Table 3:** End-force combinations of the independent test samples

Case	*F*1 (N)	*F*2 (N)
A1	158.45	214.49
A2	172.66	239.20
A3	194.80	164.42
A4	197.00	244.22
A5	225.17	169.62
B1	156.80	247.37
B2	168.35	190.90
B3	184.17	226.20
B4	210.58	157.05
B5	233.87	180.88

**Table 4:** Comparison of optical parameters fitted from the measured and TLNN-predicted profiles for the five independent forward-prediction test cases The differences are defined as TLNN-predicted values minus measured values.

Case	Center RoC from measured profile (m)	Center RoC from TLNN-predicted profile (m)	ΔCenter RoC (m)	Image distance from measured profile (m)	Image distance from TLNN-predicted profile (m)	ΔImage distance (mm)
A1	590.11	590.28	+0.17	5.6149	5.6168	+1.9
A2	539.21	539.25	+0.04	5.0783	5.0787	+0.4
A3	623.16	622.61	-0.55	5.9687	5.9639	-4.8
A4	509.10	509.06	-0.04	4.7659	4.7656	-0.3
A5	575.55	575.08	-0.47	5.4603	5.4553	-5.0

**Table 5:** Algorithm parameters used in the TLNN-DE inverse optimization

Parameter	Value
Algorithm name	soea_DE_targetToBest_1_L_templet
Population size	100
Maximum generations	50
Mutation factor	0.6
Crossover probability	0.9
